# Development of Clinical Rating Criteria for Tests of Lumbopelvic Stability

**DOI:** 10.1155/2012/803637

**Published:** 2011-12-29

**Authors:** Margaret A. Perrott, Tania Pizzari, Mark Opar, Jill Cook

**Affiliations:** ^1^Musculoskeletal Research Centre, La Trobe University, Bundoora, VIC 3086, Australia; ^2^School of Physiotherapy, La Trobe University, Melbourne, VIC 3086, Australia; ^3^School of Phsyiotherapy, La Trobe University Melbourne, VIC 3086, Australia; ^4^Faculty of Medicine, Nursing and Health Sciences, Monash University, Melbourne, VIC 3199, Australia

## Abstract

*Background*. Lumbopelvic stability (LPS) is regarded as important for injury prevention, yet there are few reliable or valid tests that can be used in the clinical assessment of LPS. Three dynamic functional tests were identified that assess LPS in multiple planes of motion: dip test (DT), single leg squat (SLS), and runner pose test (RPT). Existing rating criteria for SLS have limited reliability and rating criteria for DT and RPT have not been established. *Objective*. To develop rating criteria for three clinical tests of LPS. *Design*. Qualitative research: focus group. *Method*. A focus group of five expert physiotherapists used qualitative methods to develop rating criteria for the three clinical tests. *Results*. Detailed rating criteria were established for the three tests. Each key factor considered important for LPS had characteristics described that represented both good and poor LPS. *Conclusion*. This study established rating criteria that may be used to clinically assess LPS.

## 1. Introduction

Lumbopelvic stability (LPS) is a highly complex integrated function involving control of many segments of the body [1]. Clinically, there is a perception that LPS is an essential component of injury prevention and training of such stability is thought to aid recovery from injury and improve performance [2]. Despite this, few reliable or valid clinical tests of LPS have been identified, and attempts to establish their reliability may have been hampered by a lack of suitable rating criteria [3].

It is important to establish reliable and valid clinical tests as deficits in factors that contribute to a stable lumbopelvic region have consequences for individuals. Deficits in muscle endurance [4, 5], motor control [6–11], muscle size [12, 13], and strength [6, 14–17] have all been associated with pain or injury. 

The ability to properly assess the stability of an individual depends on the formulation of an adequate definition of LPS. Stability of the lumbopelvic region has been described by a number of authors. Bergmark proposed a mechanical engineering description of stability [18]. This stated that stability exists when the forces and the resulting moment acting on a structure maintain the structure in a state of equilibrium. This description sets the foundation of stability but has some limitations for clinical application. 

Stability of the lumbopelvic region has been described by other authors in broad terms [1, 19]. These descriptions involve control of position and motion of the trunk, pelvis, and thigh requiring that the region be in correct alignment but allowing for that fact that movement does occur. These descriptions of stability of the region appear to be clinically useful. 

The purpose of this study was to examine stability of the lumbopelvic region rather than stability of individual segments or within individual segments. However, models of intervertebral stability and intrapelvic stability add to our understanding of regional stability. Panjabi [20, 21] made an important contribution by describing a neutral zone with minimal muscle activity around a neutral posture. Stability between vertebrae was provided by an interaction between active, passive, and neural systems. Likewise, within the pelvis, stability is maintained by passive structures [22, 23] and active muscular forces [24].

Other researchers have investigated the active muscular and neural stability systems of the lumbar spine making important contributions to understanding the stability of the lumbopelvic region. Desirable patterns of muscle activation of transverse abdominus and multifidus have been described [8, 25–29]. Less desirable motor patterns have been described as substitution strategies [26, 28, 30, 31]. These may include excessive use of other muscles and pelvic tilt that may increase or decrease lumbar lordosis. 

By synthesising the literature in this area, LPS could be defined as the ability of an individual to attain and then maintain optimal body segment alignment of the spine (lumbar and thoracic), the pelvis, and the thigh in both a static position and during dynamic activity. Stability is attained and maintained by passive structures and with optimal muscle recruitment patterns, that is, without substitution strategies. 

The clinical importance of LPS highlights the need for valid clinical tests of LPS. To examine LPS, it has been recommended that assessment should be performed in an upright position with evaluation of dynamic trunk control over the weightbearing leg, in all planes of motion: sagittal, frontal, and transverse planes [1]. This type of assessment is in keeping with the above definition of LPS where the alignment of segments of the body during dynamic activity is the key feature of interest. 

**Figure 1:**
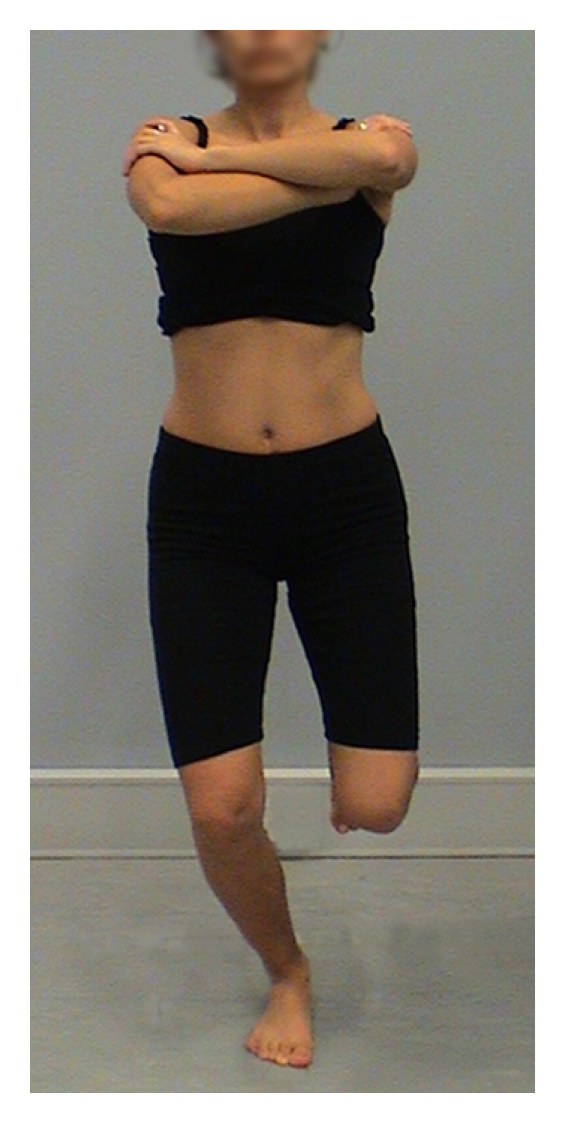
Single leg squat.

**Figure 2:**
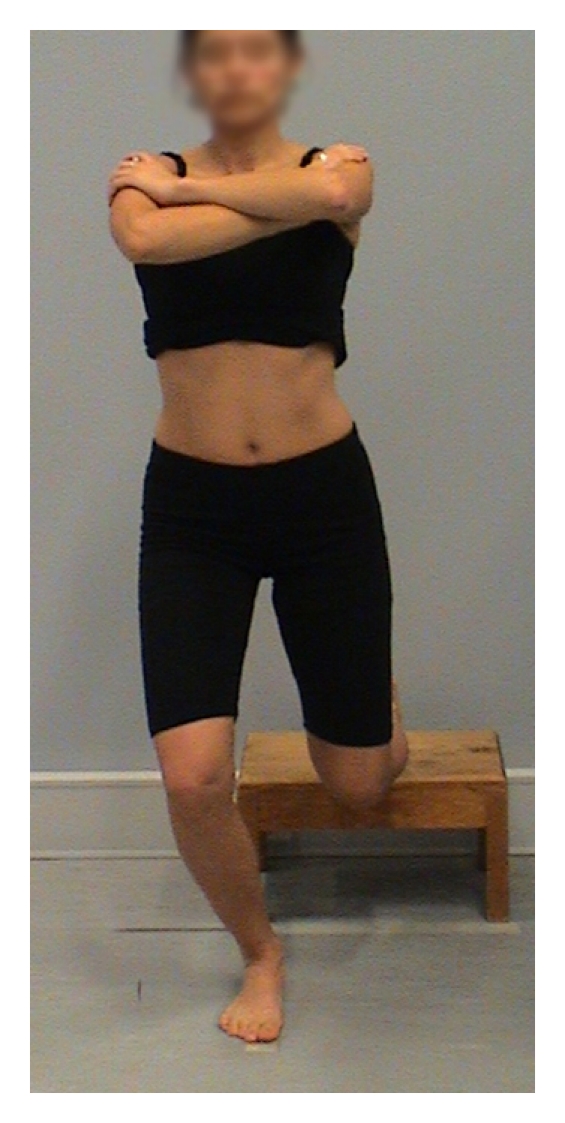
Dip test.

**Figure 3:**
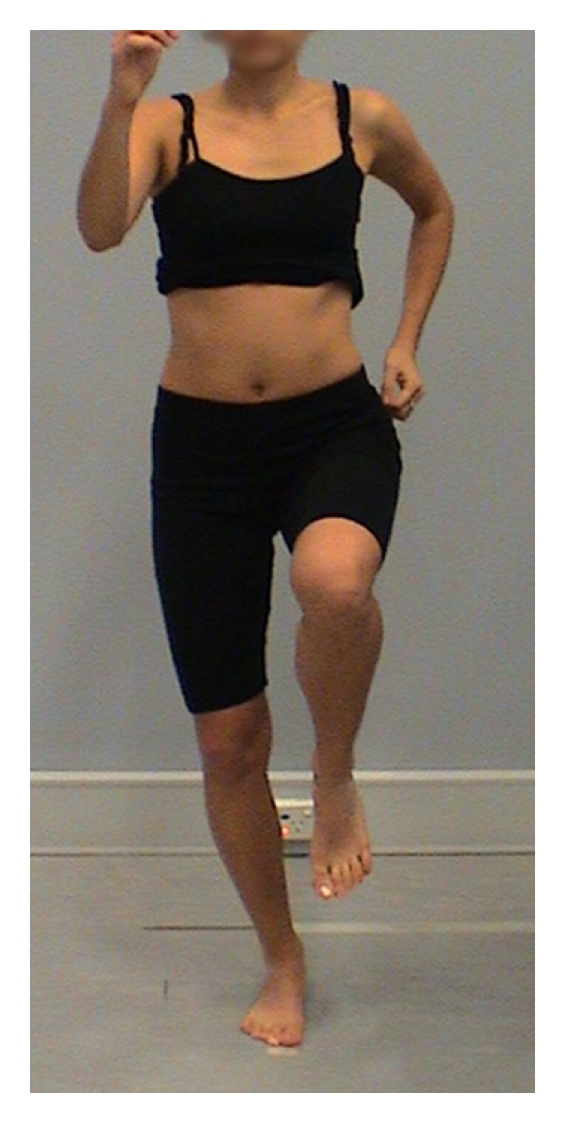
Runner pose test.

A number of dynamic tests of LPS have been reported in the literature. These include the single leg stand [32–34], single leg squat [32, 35–38], dip test [33], lateral step down [3, 36], anterior step down [36], hop test [33], and runner pose test [33, 37]. Tests of individual planes of movement were also identified [1, 36]. Several of these tests lack aspects of validity; single leg stand test, anterior step down, and lateral step down predominantly assess pelvic, hip, and knee movement in the frontal plane, and hop test has a ballistic nature that may make assessment of optimal muscle recruitment and substitution strategies difficult to achieve and to assess clinically. Of the reported clinical tests of LPS, the single leg squat (SLS, Figure 1), dip test (DT, Figure 2), and runner pose test (RPT, Figure 3), appear to assess body segment alignment of the trunk, pelvis, and thigh in multiple planes making them suitable as tests of LPS. 

For DT and RPT, assessment of performance has been described but there are no agreed rating criteria for these tests. For SLS, rating criteria were used in three studies [3, 32, 39]. Two of these studies assessed the magnitude of deviation from neutral alignment and degree of movement oscillation on a four-point rating scale with movement described as having excessive, moderate, small, or no deviation [3, 39]. Limited agreement in rating was reported using the criteria, and it was suggested that lack of explicit criteria for rating performance hampered reliability [3]. The rating criteria used by DiMattia et al. (2005) [32] only examined hip and knee alignment, so the use of these criteria for the clinical rating of LPS is limited. 

Since the three clinical tests lack valid rating criteria, it is not currently possible to use them to reliably assess LPS. Therefore, the aim of this project was to develop clinical rating criteria for the SLS, DT, and RPT for rating LPS.

## 2. Methods

A qualitative research method was used to develop rating criteria for the three tests. Material related to LPS was presented to a focus group of expert physiotherapists, discussion on characteristics of good and poor LPS was held, themes related to assessment of these characteristics were clarified, and rating criteria were qualitatively developed.

Five physiotherapists were recruited for a focus group to develop rating criteria for SLS, DT, and RPT. These independent physiotherapists had not participated in the study design and were not authors of this study. The physiotherapists were included as participants if they were accredited as expert musculoskeletal or sports physiotherapists with the national physiotherapy association and were familiar with the use of lumbopelvic stability tests. The physiotherapists were working with sports participants competing at either international or national level in the following sports: gymnastics, softball, track and field, triathlon, or Australian football. They had been practising as physiotherapists for between 6 years and 28 years (mean 14.0 yrs). Approval for the project was obtained from the Human Ethics committee of La Trobe University (application number: 07-136) and informed consent was obtained from all participants. 

The focus group met for 2 hours to develop the rating criteria. A preliminary discussion schedule of factors that might be indicative of either good or poor performance was presented to the group by the chief investigator, a sports physiotherapist with 26-year experience. This schedule included the above definition of LPS, the protocol for performing the three tests, and abnormal movement patterns of the trunk, pelvis, and hip in all three planes. The chief investigator was familiar with the LPS literature, had developed the definition of LPS, and facilitated discussion within this framework. The focus group was asked to develop criteria that would clearly rate a performance of SLS, DT, and RPT as good or poor on a three-point rating scale with an intermediate category of neither good nor poor [35]. The group viewed video footage of sample performances on DVD and discussed the factors they considered important for performance of the tests. Key stability themes and characteristics of good and poor stability were identified. 

The discussion, recorded in written notes, was read back to the group immediately for further consideration and clarification of the key stability themes. Time was allowed for discussion of the themes with contributions from each member. The themes were divided by the group into separate factors important for stability with representative characteristics of both good and poor stability. The factors and characteristics were confirmed by the group. To enhance rigour and trustworthiness [40], the rating criteria were sent via email within three days of the meeting to the focus group members to be checked for accuracy. Members of the group were invited to correct errors and suggest changes. All members confirmed the accuracy of the criteria and no changes were suggested.

## 3. Results

Agreement was reached by the focus group on rating criteria for the three tests (Tables 1, 2, and 3). The rating criteria included key factors important for LPS: five for SLS and DT and four for RPT. The factors were similar for SLS and DT: overall impression, weight transfer (SLS) or weight distribution (DT), lumbar spine and pelvic alignment, leg alignment, and foot alignment. The factors differed only slightly for RPT with foot alignment not included and evaluation of the entire trunk included, rather than just the lumbar spine. The factors for RPT were overall impression, weight distribution, trunk alignment, and pelvic alignment. 

Each of the key factors for the tests had descriptive performance characteristics that represented both good and poor stability. The combination of performance characteristics would be used to rate an individual's LPS on each of the tests. A clinical rating would be based on whether an individual predominantly had good performance characteristics or predominantly poor performance characteristics. If an individual did not clearly fit into either of these two categories, then they would be rated as having neither good nor poor stability. The combined performance characteristics would identify the LPS of an individual in one of three categories: good, poor, or neither good nor poor LPS.

## 4. Discussion

This study used qualitative methods to develop detailed rating criteria for three clinical tests of LPS: single leg squat, dip test, and runner pose test. This is the first time that detailed rating criteria have been developed for DT and RPT. 

Use of a focus group to develop rating criteria for the tests of LPS was a pragmatic way to collect views of a sample of expert physiotherapists. This method was chosen for two reasons. First, it was a method that could draw on the interaction within the group to enhance the results, making them more dynamic than the results that might be gained by the use of individual interviews or questionnaires [41]. Second, it was economical in terms of time and cost.

To ensure the integrity of the results of qualitative studies, it is important that the phenomenon being studied is presented accurately [40]. The definition of LPS was derived from the stability literature, and discussion to develop the rating criteria was held from this perspective. 

To develop rating criteria, a pool of potential variables should be considered as the first step [42]. The rigour of this process was enhanced by using multiple sources of information [43]. The focus group used the preliminary discussion of abnormal movement patterns of the trunk, pelvis, and hip described in previous studies of single leg squat [3, 32, 39], their clinical experience, and the DVD examples of performance of the tests to identify key indicators of good or poor performance of the LPS tests. The chief investigator ensured that all potential performance characteristics from the LPS literature were discussed. 

The second step in the process of developing rating criteria is to decide the final variables to be included [42]. This was accomplished by discussion of the key themes that had been identified: overall impression, weight transfer or distribution, and alignment of the lumbar spine or trunk, pelvis, leg, and foot. Characteristics of good and poor performance were described. For rating criteria to have construct validity, the domains of interest must be adequately assessed [44]. The criteria developed in this study comprehensively covered all aspects thought to be important for LPS and built on previous rating criteria for SLS [3, 32, 39]. The criteria developed in this project were also very similar to those developed for step-down test [45]. Thus, there is preliminary evidence for construct validity for the rating criteria.

A third step in the development of rating criteria has been described as giving a relative weighting or emphasis to the final items included [42]. This was not done in this study. The rating criteria were designed for all items to be combined to achieve the overall rating of LPS rather than to individually rate items or subcategories. This was done to maintain the clinical utility of the criteria as tests that require this may not be as easy or quick to administer [46, 47]. In addition, experienced raters are likely to combine key observations to make an automatic rating [48] based largely on the overall impression of movement quality, and this method of rating is commonly used by physiotherapists [45].

The trustworthiness of a qualitative research process relies on credibility [49]. Credibility is enhanced by the experience and qualifications of the investigators, member checks, and similarity to previous studies [40]. This study met each of these three principles. First, the members of the focus group were experienced in examining LPS. These well-qualified clinicians with a mean of 14-year experience in a wide variety of sports would be expected to be able to identify good and poor performance characteristics of LPS. Second, member checks were performed during and after the focus group discussion to confirm the themes that were developed. Each member had the opportunity to individually comment on or suggest changes to the criteria. This mitigated the possible effect of any one member of the group dominating the outcome and ensured that the criteria truly represented the perceptions of LPS of each member of the group. Third, the discussion themes and final rating criteria shared some characteristics with rating criteria used in other studies of SLS with the inclusion of assessment of trunk, pelvis, and thigh [3, 32, 39]. The rating criteria developed in this project also included assessment of foot alignment for SLS and DT. Foot function may influence performance via the closed kinetic chain used in these tests. The lack of evaluation of foot function was discussed as a limitation in a study of the step down test by Crossley et al. (2011) [45]. For these reasons, it is credible that these criteria do represent the characteristics of good and poor stability. 

The trustworthiness of a qualitative research process also depends on transferability [49], whether the rating criteria are transferable or generalizable to a range of individuals or whether they would have limited application. They are transferable and suitable for the orthopaedic assessment of active male and female adults. The validity of their use as a screening tool, as a means of identifying injury, or a means to assess timing of a return to sport remains to be investigated in prospective injury studies. Their use in other populations such as older adults, elite athletes, children, and populations with neurological conditions also remains to be investigated. Other confounding factors may be introduced in these populations that would reduce the clinical utility of these rating criteria. 

Qualitative focus group studies have some disadvantages and these are acknowledged as limitations. Disadvantages relate to the role of the facilitator, accurate recording of the discussion, and management of issues of dissent [41]. First, results can depend on the skill of the group facilitator to generate unbiased discussion without influencing the group to arrive at a predetermined conclusion. The extent to which the group may have been influenced by the facilitator cannot be determined. Second, inaccuracy of recording of the discussion may influence the results. However, as discussed above, the notes were read back to the group and each group member had an opportunity to clarify and correct the results, so it is expected that this limitation would be reduced. Third, dissent within a focus group may bias results if less confident members of a group are unable to raise objections. As already discussed, each member was individually able to raise objections by email, it is expected that this disadvantage was reduced. 

## 5. Conclusions

The present study has developed detailed rating criteria that may be used by clinicians to rate the LPS of individuals using three clinical tests. Single leg squat, dip test, and runner pose test each have key factors that are important in the assessment of LPS. Performance characteristics of good and poor LPS have been identified. In terms of a clinical reasoning process, it is likely that an intervention to address the LPS of an individual rated as having poor LPS would be a priority. Intervention would be less of a priority for those rated as having neither good nor poor stability and may be unnecessary for those rated as having good LPS.

## Figures and Tables

**Table 1:** Rating criteria for single leg squat: good and poor stability.

*Single Leg Squat*	
Good	Poor

1. Overall Impression	
Smooth, good-quality movement General control Controlled change-over between repetitions Ease of movement	Staggered movement Increased speed to attempt to control movement Effort to control movement Trunk “wobble”

2. Weight Transfer	
Minimal translation of centre of mass Upright trunk	Discernible translation of centre of mass Trunk leaning forward or to side Extended time to transfer

3. Lumbar Spine & Pelvic Alignment	
Minimal movement in all three planes Frontal plane: ASIS level Sagittal plane: minimal A-P tilt, rotation Lateral view: stable lordosis, minimal trunk flexion	Discernible movement with pelvis tilting up or down, rotating toward or away from weightbearing leg, tilting in anterior or posterior direction Lumbar lordosis increasing or trunk flexion occurring

4. Leg Alignment	
Minimal movement out of the starting plane of movement. This takes into account the alignment of the limb, influenced by pelvic width, and Q angle at the knee	Discernible movement out of the starting plane of movement

5. Foot Alignment	
Neutral foot position—remains stable during movement	Excessive pronation of foot during squat descent Externally rotated starting position of lower leg/foot

**Table 2:** Rating criteria for dip test: good and poor stability.

*Dip Test*	
Good	Poor

1. Overall Impression	
Smooth, good-quality movement General control Controlled change-over between repetitions	Staggered movement Increased speed to attempt to control movement Effort to control movement Trunk “wobble”

2. Weight Distribution	
Minimal weight on back leg Back leg remains oriented in the sagittal plane (i.e. no movement in frontal plane) Upright trunk	Excessive weight on back leg Abduction of back leg Trunk leaning forward or to side

3. Lumbar and Pelvic Alignment	
Minimal movement in all three planes Frontal plane: ASIS level Sagittal plane: minimal A-P tilt, rotation Lateral view: stable lordosis, minimal trunk flexion	Discernible movement with pelvis tilting up or down, rotating toward or away from weightbearing leg, tilting in anterior or posterior direction

4. Leg Alignment	
Minimal movement out of the starting plane of movement. This takes into account the alignment of the limb, influenced by pelvic width, and Q angle at the knee	Discernible movement out of the starting plane of movement

5. Foot Alignment	
Neutral foot position—remains stable during movement	Excessive pronation of foot during squat descent Externally rotated starting position of lower leg/foot

**Table 3:** Rating criteria for runner pose test: good and poor stability

*Runner Pose Test*	
Good	Poor

1. Overall Impression	
Smooth, good-quality movement General control	Jerky movement Effort to control movement Excessive trunk movement

2. Weight Distribution	
Minimal translation of centre of mass	Inability to maintain centre of mass over weightbearing leg

3. Pelvic Alignment	
Hip dissociation from pelvis—minimal pelvic movement Minimal A-P movement (anterior pelvic tilt) Minimal tilt in frontal plane (ASIS level)	Discernible movement of pelvis with the hip—no dissociation Discernable tilt in frontal plane

4. Trunk Alignment	
No rotation of trunk Trunk upright	Trunk rotation Forward flexion of trunk Trunk locked in extension
